# Intraventricular Hemorrhage: Risk Factors and Association With Patent Ductus Arteriosus Treatment in Extremely Preterm Neonates

**DOI:** 10.3389/fped.2019.00408

**Published:** 2019-10-22

**Authors:** Ijab Khanafer-Larocque, Amuchou Soraisham, Amelie Stritzke, Essa Al Awad, Sumesh Thomas, Prashanth Murthy, Majeeda Kamaluddeen, James N. Scott, Khorshid Mohammad

**Affiliations:** ^1^Section of Neonatology, Department of Pediatrics, University of Calgary, Calgary, AB, Canada; ^2^Section of Neonatology, Department of Pediatrics, University of Alberta, Edmonton, AB, Canada; ^3^Departments of Diagnostic Imaging and Clinical Neurosciences, University of Calgary, Calgary, AB, Canada

**Keywords:** intraventricular hemorrhage, patent ductus arteriosus, preterm, brain injury, neonate, care bundle

## Abstract

**Objectives:** To assess maternal and neonatal risk factors for intraventricular hemorrhage (IVH). To examine the association of patent ductus arteriosus (PDA) and its treatment, with IVH and its severity.

**Study design:** In this retrospective cohort study, we included preterm neonates born at <29 weeks, admitted to a tertiary level III Neonatal Intensive Care Unit in Calgary, Canada, between 2013 and 2016, who had a head ultrasound in the first 7 days of life. A subset analysis included neonates who also had cardiac ultrasound in the first 3 days of life.

**Results:** Of the 495 neonates, 121 (24.4%) had IVH of any grade and 48 (9.7%) had severe IVH. Identified risk factors were small birth gestation and weight, lack of antenatal corticosteroids, maternal chorioamnionitis, Apgar score <5 at 5 min, umbilical cord pH < 7, respiratory distress syndrome, early onset sepsis, hypercapnia, pCO_2_ fluctuations, prolonged intubation, inhaled nitric oxide, inotropes or normal saline boluses, metabolic derangements, opioids infusions, and bicarbonate/THAM therapy. In a primary analysis of the total cohort, when the decision to treat a PDA was used as a surrogate marker of its clinical significance, a PDA requiring treatment was associated with a higher risk of IVH. There was no significant difference in the incidence of IVH between neonates with early treatment of a clinically significant PDA compared to late, however early indomethacin treatment was associated with reduced severity of IVH. In the subset analysis, the presence of a hemodynamically significant PDA (hs-PDA) was not associated with a higher probability of IVH. Of those with severe IVH, 18 (55%) had a hs-PDA; this is clinically but not statistically significant.

**Conclusions:** Identified risk factors should be the target of IVH reduction bundles. Early indomethacin treatment for a clinically significant PDA may reduce IVH severity.

## Introduction

Extremely preterm neonates (<29 weeks) have an increased risk of intraventricular hemorrhage (IVH) which is a multifactorial medical complication. A hemodynamically significant PDA (hs-PDA) can cause fluctuations in cerebral blood flow (1–3), which are associated with IVH (2–5), possibly in an ischemia-reperfusion injury model (5, 6).

### IVH

In the neonatal literature, the severity of IVH is graded from I to IV, depending on its ultrasound imaging appearance. Severe IVH refers to equal or greater than Papile's or Volpe's Grade III. Papile (7) defines Grade III as IVH with ventricular dilatation, and Grade IV as IVH with intraparenchymal hemorrhage. Volpe's (8) Grade III is an IVH that takes up >50% of the ventricular area on parasagittal views and is usually associated with ventricular dilatation. Volpe resists the use of the Grade IV term because it infers progression from a Grade III IVH, which is not the case, but in his equivalent of Papile's Grade IV, he refers to periventricular echodensity or periventricular/parenchymal hemorrhagic infarction (9). Approximately half of IVH occurs in the first 24 h, and 80–90% within the first 72 h of life (10).

The incidence of all grades of IVH in the extremely preterm population varies between 31 and 36% (11–13), while the incidence of severe IVH varies between 10 and 17% (11–16). The significance of IVH pertains to its detrimental effect on long-term neurodevelopmental outcome, which despite being worse in severe IVH, is not negligible in mild IVH (17). Of additional concern, neonates with severe IVH have increased mortality (11). Severe IVH may lead to post-hemorrhagic ventricular dilatation (PHVD), seizures, cerebral palsy, developmental delay, deafness, and blindness (10, 11). In a large cohort study, Synnes et al. reported that brain injury on head ultrasound was the strongest predictor for significant neurodevelopmental impairment or death, with an odds ratio of 4.5 (95% CI 3.47, 5.84) (16).

### PDA

The patent ductus arteriosus (PDA), a shunt between the pulmonary artery and the aorta, is essential for fetal circulation. It normally constricts shortly after birth in term neonates. However, spontaneous ductal closure by the seventh day of life occurs in only 13–36% of preterm neonates born at less 29 weeks; the younger the gestation, the higher the risk of ductal persistence (18). After birth, a PDA may result in shunting of blood from the left to the right circulation, which can compromise perfusion to various organs. Studies have shown associations between a hs-PDA and IVH, bronchopulmonary dysplasia (BPD), necrotizing enterocolitis (NEC), cardiac failure, hypotension, pulmonary hemorrhage, and mortality (19–22).

Significant controversy remains surrounding the optimal management of a hs-PDA; there is a lack of consensus regarding which PDA to treat, when and how. Treatment options include conservative treatment, non-steroidal anti-inflammatory drugs (NSAIDs) such as indomethacin and ibuprofen, acetaminophen, or surgical ligation.

### IVH and PDA Treatment

Literature review regarding the impact of PDA treatment on the incidence of IVH is contradicting. An analysis of neonates not recruited into the PDA-TOLERATE trial (a randomized control study) does bring into focus that the more immature and unwell neonates are more likely to be excluded from such studies, which could affect the results (23). A Cochrane review from 2010 found that prophylactic treatment of PDA with indomethacin reduced any IVH (RR 0.88, 95% CI 0.80, 0.98), as well as severe IVH (RR 0.66, 95% CI 0.53, 0.82) (24). In contrast, a Cochrane review from 2011 indicated that prophylactic treatment with ibuprofen did not show a significant reduction in IVH (all grades or severity) (25). The Australian DETECT study used early ultrasound to assess the presence of a PDA in order to target treatment with indomethacin; they showed a reduction in pulmonary hemorrhage but were underpowered to detect differences in IVH (26). Given that approximately one third of neonates born at <29 weeks of gestation have a PDA that constricts spontaneously (without intervention), a selective approach to determine which neonates would actually benefit from treatment would be preferable in order to avoid the risks associated with treatment. In this study, we aimed to assess maternal and neonatal risk factors for intraventricular hemorrhage (IVH) and examine the association of patent ductus arteriosus (PDA) and its treatment, with IVH and its severity.

## Patients and Methods

### Study Design, Population, and Setting

This was a retrospective analysis of a prospectively collected cohort of all neonates born at a gestational age of <29 weeks, admitted to the Neonatal Intensive Care Unit (NICU) at Foothills Medical Center in Calgary, Canada, between January 1, 2013 and December 31, 2016, who had a head ultrasound in the first 7 days of life. We excluded neonates with major congenital or chromosomal anomalies, antenatally diagnosed IVH, incomplete dataset, or who died prior to obtaining a head ultrasound. A secondary analysis examined the subset of neonates who had a Targeted Neonatal Echocardiogram (TNE) in the first 72 h of life.

### Ethics and Data Source

This study was approved by the Conjoint Health Research Ethics Board (CHREB) at the University of Calgary and the consent was waived. Data was collected prospectively, using predefined variables, as part of a local quality improvement project aiming at IVH prevention (“Drive to Zero”), also approved by CHREB (REB14-1466). Data collected included demographic characteristics, head and cardiac imaging, as well as other morbidities. The database analysis allowed us to determine the incidence and severity of IVH, as well as the incidence of untreated and treated PDA in our cohort. We were then able to examine the incidence and severity of IVH between neonates treated for PDA and those without PDA treatment.

### Local Guidelines

“Drive to Zero” is a Calgary-based project that aims to reduce IVH in the preterm population. The “Intraventricular Hemorrhage and Brain Injury Prevention Package” (IVH-BIPP) is a care bundle that targets premature neonates of ≤ 30^+6^ weeks gestation in the first 72 h of age. This bundle consists of recommendations on respiratory and hemodynamic management, lighting, noise, thermoregulation, skin care, minimal handling, positioning, and obtaining vascular access. This document is available under Supplementary Data online and as a Canadian Pediatric Society (CPS) position statement (27).

According to local guidelines, neonates born at <32 weeks of gestation, or with a birth weight of <1,500 g, have a head ultrasound between days 5–7 of life, or earlier, if clinically indicated, to assess for IVH. All images are reviewed and scored blindly by an independent neuroradiologist (JNS), who is unaware of the neonate's clinical course using a standard template (available under Supplementary Data). Local guidelines also suggest an echocardiogram or TNE to assess for a PDA in neonates born at <30 weeks in the first 2–7 days of life. Upon diagnosis of a hs-PDA, the decision to treat and modality of treatment rests with the most responsible physician.

The IVH-BIPP guided clinical decisions such as the administration of normal saline boluses and/or inotropes, extubation, and reintubation. The use of iNO was directed by the requirement of a high FiO_2_ with evidence of Persistent Pulmonary Hypertension, and not Respiratory Distress Syndrome (RDS).

### Operational Definitions

The “IVH window” refers to the first 72 h of life, during which there is the highest risk of IVH. For this study, we defined “Mild IVH” as IVH grades I or II, and “severe IVH” as IVH grades III or IV, moderate to severe post-hemorrhagic ventricular dilation, and/or periventricular leukomalacia.

For this study, a hs-PDA was defined as a PDA of 1.5 mm or more in diameter at its narrowest point with complete or predominantly left to right shunting, and showing a low velocity flow on Doppler interrogation, defined as <1.5 m/s, or the presence of signs of left volume loading with a LA:Ao ratio of >1.5. Echocardiographic findings were further classified into four phenotypes: (a) moderate to large shunt volume—if the PDA size was >1.5 mm, the flow pattern was growing or pulsatile, and the shunt direction was left to right or mostly left to right with evidence of volume overloading of the left side; (b) small shunt volume—if the size was ≤1.5 mm, the flow pattern was restricted, and the shunt direction was left to right; (c) non-PDA physiology—PDA with complete left to right shunt or bidirectional with a pulsatile pattern; and (d) no PDA. The echocardiographic data was reviewed by neonatologists trained in performing and reading TNE who were blinded to the head imaging findings.

The timing of PDA treatment was considered early if it was given within the first 72 h of life, and late if it was given between 72 h and 14 days. Surgical ligation was considered for infants with a hs-PDA, which failed to close after 2–3 courses of medical treatment or contraindication to medical treatment.

In the first 72 h of life, any infant requiring reintubation was counted as an extubation failure, pCO_2_ fluctuation was defined as the recording of at least one hypocapnea (arterial or capillary pCO_2_ < 35 mmHg) and at least one hypercapnea (arterial or capillary pCO_2_ > 60 mmHg), hypoglycemia as any blood glucose level <2.6 mmol/L, hyperglycemia as any blood glucose level >10 mmol/L, hypothermia as any temperature <36 C, hyperthermia as any temperature higher than 37.5 C, acidosis as any arterial or capillary blood pH < 7.20, and alkalosis as any arterial or capillary pH > 7.45. Early onset sepsis was defined as a positive blood culture in the first 48 h. At our center, the most common agents used for sedation are morphine or fentanyl infusions.

### Statistical Analysis

Statistical analysis was performed with Stata 14. Descriptive statistical analysis was performed. We compared the maternal and neonatal characteristics between infants with no IVH, mild IVH and severe IVH. We used Chi Square test or Fisher exact test for comparing the categorical variables and analysis of variance for comparing the continuous variables. The tests used were two-sided and significance was defined as *p* < 0.05.

## Results

### IVH

Of the 500 eligible neonates admitted during the study period, 495 neonates were included. Five infants were excluded due to incomplete data or death prior to head ultrasound. Of the 495 neonates, 121 (24.4%) had IVH of any grade, and 48 (9.7%) had severe IVH. Grade I and Grade II accounted for 34 (28%) and 39 (32%) cases, respectively, while Grade III and Grade IV accounted for 6 (5%) and 42 (35%) cases, respectively. The incidence of IVH was reduced with increasing gestational age. The incidence of IVH (any grade) at 23, 24, 25, 26, 27, and 28 weeks was 30.4, 48.6, 30.8, 25, 18.7, and 11.9%, respectively. The incidence of severe IVH followed a similar trend. At 23, 24, 25, 26, 27, and 28 weeks, 8.7, 21.6, 13.8, 6.5, 7.3, and 5.2% had severe IVH, respectively.

The neonates in the severe IVH group had less antenatal steroids exposure, but increased exposure to clinical chorioamnionitis, as compared to the no IVH group. The neonates in the IVH group (both mild and severe) were more immature, had smaller birth weights, and had a lower 5-min Apgar score compared to those without IVH (Table 1). Furthermore, neonates in the severe IVH group had more RDS, and were more likely to be ventilated and to receive inhaled nitric oxide during the first 72 h of age. Hypercapnia and pCO_2_ fluctuations, as well as the use of normal saline boluses and inotropes, were more common in the mild and severe IVH groups, as compared to the no IVH group. Neonates in the IVH group had more hyperglycemia, hypothermia and early onset sepsis. The early neonatal mortality rate was significantly higher in the mild and severe IVH groups, as compared to the no IVH group (Table 1).

**Table 1:** Comparison of demographic characteristics, interventions in the first 72 h and morbidity of neonates without IVH and those with IVH.

	**No IVH (*n* = 374)**	**Mild IVH (*n* = 73)**	**Severe IVH (*n* = 48)**	***p*-value**
**Maternal characteristics**, ***n*** **(%)**				
Antenatal steroids	320 (85.6)	55 (75.3)	32 (66.6)	0.001
Rupture of membrane >24 h	81 (21.6)	15 (20.5)	8 (16.6)	NS^*^
Chorioamnionitis	63 (16.8)	14 (19.1)	14 (29.1)	<0.001
Vaginal delivery	208 (55.6)	49 (67.1)	32 (66.7)	0.08
**Neonatal characteristics**				
Gestational age, mean ± SD (weeks)	26.4 ± 1.5	25.5 ± 1.53	25.5 ± 1.57	<0.001
Birth weight, mean ± SD (g)	893 ± 236	816 ± 227	805 ± 217	0.004
Male, *n* (%)	192 (51.3)	37 (50.6)	30 (62.5)	NS
Small for gestational age, *n* (%)	48 (12.8)	7 (9.5)	4 (8.3)	NS
Multiple gestations, *n* (%)	103 (27.5)	23 (31.5)	17 (35.4)	NS
Apgar score <5 at 5 min, *n* (%)	71 (18.9)	12 (16.4)	17 (35.4)	0.01
Umbilical cord pH < 7, *n* (%)	11 (2.9)	2 (2.7)	3 (6.2)	0.03
Delayed cord clamping, *n* (%)	145 (38.8)	23 (31.5)	14 (29.1)	NS
Outborn, *n* (%)	32 (8.6)	9 (12.3)	9 (18.7)	0.07
**Respiratory**, ***n*** **(%)**				
Respiratory distress syndrome	269 (71.9)	70 (95.8)	47 (97.9)	<0.001
Extubated within 72 h	137 (36.6)	25 (34.2)	8 (16.6)	0.02
Extubation failure	27 (7.2)	8 (10.9)	8 (16.6)	0.06
Pneumothorax	16 (4.3)	1 (1.3)	2 (4.1)	NS
Inhaled nitric oxide treatment	52 (13.9)	12 (16.4)	14 (29.1)	0.02
Hypocapnea	149 (39.8)	22 (30.1)	24 (50)	0.08
Hypercapnea	113 (30.2)	38 (52)	32 (66.6)	<0.001
pCO2 fluctuation	35 (9.4)	13 (17.8)	17 (35.4)	<0.001
**Cardiovascular**, ***n*** **(%)**				
Normal saline bolus use	87 (23.2)	39 (53.4)	28 (58.3)	<0.001
Inotrope use	35 (9.3)	20 (27.4)	23 (47.9)	<0.001
Dopamine	35 (9.3)	14 (19.1)	18 (37.5)	<0.001
Dobutamine	32 (8.5)	16 (21.9)	18 (37.5)	<0.001
**Metabolic**, ***n*** **(%)**				
Hypoglycemia	124 (33.1)	26 (35.6)	18 (37.5)	NS
Hyperglycemia	43 (11.5)	19 (26)	9 (18.7)	0.02
Hypothermia	21 (5.6)	4 (5.4)	5 (10.4)	0.03
Hyperthermia	41 (10.9)	11 (15)	7 (14.5)	0.05
Acidosis	111 (29.6)	39 (53.4)	35 (72.9)	<0.001
Alkalosis	21 (5.6)	5 (6.8)	3 (6.2)	NS
Bicarbonate/THAM therapy	14 (3.7)	7 (9.5)	12 (25)	<0.001
**Other**, ***n*** **(%)**				
Sedation	39 (10.4)	16 (21.9)	26 (54.1)	<0.001
Early onset sepsis	6 (1.6)	4 (5.4)	3 (6.2)	0.04
Mortality in first week	10 (2.7)	6 (8.2)	16 (33.3)	<0.001

* *NS, not significant*.

### PDA

In our total cohort of 495 neonates, 198 neonates (40%) had at least one intervention for PDA treatment during their NICU stay. Medical treatment was received by 195 neonates (39%), of whom 18 (9%) received early ibuprofen and 35 (18%) received early indomethacin. Surgical ligation was performed in 43 (9%) neonates (Table 2).

**Table 2:** PDA treatment of infants without IVH and those with IVH.

**PDA treatment, *n* (%)**	**No IVH (*n* = 374)**	**Mild IVH (*n* = 73)**	**Severe IVH (*n* = 48)**	***p*-value**
**Medical treatment**				
Early (<72 h)	36 (9.4)	16 (21.9)	1 (2.1)	0.004
Anytime	128 (34.2)	47 (64.4)	20 (41.7)	<0.001
**Ibuprofen**				
Early	13 (3.5)	4 (5.5)	1 (2.1)	NS
Anytime	77 (20.6)	26 (35.6)	11 (22.9)	0.02
**Indomethacin**				
Early	23 (6.1)	12 (16.4)	0	0.02
Anytime	67 (17.9)	25 (34.2)	10 (20.8)	0.007
**Surgical, anytime**	29 (7.8)	9 (12.3)	5 (10.4)	NS
**Medical and/or surgical, anytime**	127 (34)	49 (67.1)	22 (45.8)	<0.001

^*^Some infants may have received both indomethacin and ibuprofen.

Of the 495 neonates, 258 (52%) had a cardiac ultrasound in the first 72 h (Table 3). The median time at the first cardiac ultrasound was 31 h (interquartile range 10–50 h). Infants who had early cardiac ultrasound were smaller in gestational age (25.7 ± 1.58 vs. 26.7 ± 1.33 weeks, *p* < 0.001) and birth weight (804 ± 214 vs. 949 ± 235 g, *p* < 0.001), as compared to infants who did not have early cardiac ultrasound. Of the 258 neonates, 142 (55%) had a hs-PDA, however only 32 (22.5%) of these received early medical treatment with either ibuprofen or indomethacin, and 93 (65.5%) received early and/or late medical treatment for a PDA. A number of these neonates had concomitant pulmonary hypertension, which makes PDA closure inadvisable. Interestingly, 17 neonates with a non hs-PDA and two with no PDA on initial cardiac ultrasound also received early medical treatment. These infants were likely treated based on significant clinical findings that developed after the initial cardiac imaging had already been done or based on a subsequent cardiac ultrasound that showed a hs-PDA. Seven infants received early and/or late treatment for PDA despite not having a PDA on early TNE. Of these, four had their PDA spontaneously reopen and become hemodynamically significant on later echocardiography, one was treated with indomethacin prophylactically, one had severe PPHN with decreased right ventricular contractility on a later echocardiogram (and so, was treated once pulmonary pressures normalized and right ventricular function improved), and finally, one had pulmonary hemorrhage with no documented echocardiography prior to treatment. Five out of the seven infants had an abnormal head ultrasound prior to treatment (four infants with Grade I and one with Grade II); the other two had normal head ultrasounds.

**Table 3:** Secondary analysis–subset of neonates with early cardiac imaging: PDA presence, treatment, and outcomes.

	**PDA present**		**No PDA (*n* = 31)**	***p*-value**
	**hs-PDA (*n* = 142)**	**No hs-PDA (*n* = 85)**		
**PDA treatment**, ***n*** **(%)**				
**Medical treatment, early**				
Indomethacin	24 (16.9)	11 (12.9)	1 (3.2)	NS
Ibuprofen	8 (5.6)	6 (7.1)	1 (3.2)	NS
Indomethacin or Ibuprofen	32 (22.5)	17 (20)	2 (6.5)	NS
**Medical treatment, anytime**				
Indomethacin	54 (38)	23 (27.1)	4 (12.9)	0.01
Ibuprofen	49 (34.5)	26 (30.6)	3 (9.7)	0.02
Indomethacin or Ibuprofen	93 (65.5)	44 (51.8)	7 (22.6)	<0.001
**Surgical, anytime**	16 (11.3)	10 (11.8)	2 (6.5)	NS
**Medical and/or surgical, anytime**	95 (66.9)	46 (54.1)	7 (22.6)	<0.001
**Outcomes**				
IVH (all grade)	48 (33.8)	23 (27.1)	12 (38.7)	NS
Severe IVH (grade ≥ 3)	18 (12.7)	9 (10.6)	6 (19.4)	NS
Early neonatal death (<7 days)	14 (9.9)	8 (9.4)	3 (9.7)	NS
Death/Severe IVH	26 (18.3)	11 (12.9)	8 (25.8)	NS

### IVH and PDA

In our complete cohort, we examined PDA treatment as a surrogate marker of PDA clinical significance in the groups with IVH, and without IVH. The group with IVH had more medically treated PDA (55.4 vs. 34.2%, *p* < 0.001), but there was no statistically significant difference with the early treatment of PDA (14% in the IVH group vs. 9.6%, *p* = 0.14), or treatment using ibuprofen vs. indomethacin (Table 2). However, when the IVH group was separated into mild IVH and severe IVH for further analysis (Table 2), early treatment of PDA with indomethacin was associated with decreased severity of IVH (*p* = 0.02). In the subset analysis, 142 neonates (55%) had a hs-PDA, of whom 18 (12.7%) had severe IVH. However, of those with severe IVH, 55% had a hs-PDA. Although this is clinically significant, no statistical significance was detected (Table 3).

When IVH and PDA are analyzed within the total cohort (primary analysis) and the early imaging group (secondary analysis) (Table 4), the likelihood of having any IVH was associated with needing any PDA treatment (*p* < 0.001), but no other statistically significant association was found.

**Table 4:** PDA status and IVH.

	**Primary analysis**			**Secondary analysis**		
	**Total cohort with available PDA data (*****n*** **=** **495)**		***p*-value**	**Early echo group (*****n*** **=** **258)**		***p*-value**
	**No PDA tx (*n* = 297)**	**Any PDA tx (*n* = 198)**		**No hs-PDA (*n* = 116)**	**hs-PDA (*n* = 142)**	
Any IVH, *n* (%)	50 (16.8)	71 (35.9)	<0.001	35 (30.2)	48 (33.8)	NS
Severe IVH, *n* (%)	27 (9.1)	22 (11.1)	0.46	15 (12.9)	18 (12.7)	NS

When the early cardiac imaging findings were further classified into four phenotypes (moderate to large shunt volume, small shunt volume, non-PDA physiology and no PDA), there was no significant difference seen in the incidence of IVH, however, the composite outcome of death/severe IVH was higher in the non-PDA physiology group (*p* = 0.005) (Table 5).

**Table 5:** Secondary analysis: PDA phenotypes and IVH.

	**Early echo group (*****n*** **=** **258)**				
	**No PDA (*n* = 31)**	**Small shunt volume PDA (*n* = 83)**	**Moderate to large shunt volume PDA (*n* = 83)**	**Non-PDA physiology (*n* = 61)**	***p*-value**
Any IVH, *n* (%)	11 (35.5)	23 (27.7)	29 (34.9)	20 (32.8)	NS
Severe IVH, *n* (%)	5 (16.1)	9 (10.8)	7 (8.4)	12 (19.7)	NS
Death/Severe IVH, *n* (%)	7 (22.5)	11 (13.2)	8 (9.6)	19 (31.1)	0.005

## Discussion

### IVH

We confirmed amenable antenatal and postnatal risk factors associated with IVH. Antenatally, our data highlights the importance of *in utero* growth (both in gestation and weight), and antenatal corticosteroids. Postnatally, tight pH control, maintaining normocapnea, normothermia, and normoglycemia, as well as avoiding the use of inotropes or saline boluses may be protective. In our cohort of 495 extremely preterm neonates, our rates of any IVH (24.4%) and severe IVH (9.7%) are comparatively low (11–16).

### IVH and PDA

Our local rate of intervention for PDA treatment (40%) is lower than the Canadian average of 51% (28), whereas our ligation rate is slightly above Canadian average (9 vs. 7.1%) (28).

In our total cohort, when PDA treatment was used as a surrogate marker for its clinical significance, we observed that a PDA requiring treatment was associated with a higher risk of IVH. Early treatment of a clinically significant PDA did not seem to affect the incidence of IVH, but early treatment with indomethacin was associated with decreased severity of IVH. However, this finding did not reach statistical significance in our secondary analysis, in which we used cardiac imaging in the first 72 h of life to determine clinical significance, as opposed to treatment decisions. This dissonance between our primary and secondary analysis may be because the criteria we used to determine hemodynamic significance on cardiac imaging did not in fact adequately capture clinically significant PDAs. It could also perhaps be argued that the use of the clinical judgment to treat or not to treat a PDA as a marker of its significance (primary analysis), which would be based on a bedside analysis of the neonate's entire clinical situation and not just one snapshot in time as offered by cardiac imaging (secondary analysis), provides a more accurate portrayal of PDA significance.

Our findings are consistent with the 2010 and 2011 Cochrane reviews, which showed that the use of early indomethacin was associated with a decreased severity of IVH, and that early ibuprofen did not cause a statistically significant reduction in IVH (24, 25). However, with early indomethacin, we did not see a reduction in the overall incidence of IVH (only severe IVH). We found a higher rate of death/severe IVH in neonates with a non-PDA physiology, which is most probably due to a higher mortality in infants with significant PPHN.

This analysis, which spans 4 years of data from our level III center, raises multiples practical questions that can influence our care for this fragile population. Apart from assessments for pulmonary hypertension, and cardiac contractility or filling, early cardiac imaging is usually used to assess the status of a PDA, in order to provide guidance for a treatment decision. However, if the use of early indomethacin decreases IVH, regardless of PDA status, routinely assessing the PDA in the first 72 h may be of little clinical benefit and may lead to overtreatment. The limitation of PDA assessments to only when clinically indicated will have an added benefit of minimizing handling of the baby in the IVH window. Early treatment of PDA does not seem to confer added protection to the preterm neonate. As for the use of early indomethacin, early prophylactic indomethacin decreases the incidence of IVH among extremely low birth weight neonates, whether or not they were exposed to antenatal steroids (29). The incidence of severe IVH is significantly decreased by targeted administration of indomethacin to neonates born at <29 weeks, who were not exposed to or who had received a partial course of antenatal steroids (30). Recently, prophylactic indomethacin was associated with improved survival in an out-born cohort of extremely preterm infants who were referred to a Level IV NICU (31). Of note, there was no statistical difference in survival in our cohort of neonates who had a PDA treated with early indomethacin.

The mechanism by which indomethacin affects IVH is unclear. Animal studies have suggested that it may prevent surges in cerebral blood flow, as seen in animal models with asphyxia or hypercapnia (32–34), and also by mitigating increased transport of ions and proteins across the blood-brain barrier secondary to ischemia (35), thus avoiding inappropriately elevated cerebral perfusion pressures. It may also promote germinal matrix micro-vessel maturation in newborn beagle pups (36). A recent Canadian study (37) suggested a higher incidence of spontaneous intestinal perforation among neonates who received prophylactic indomethacin, while a Cochrane meta-analysis showed a higher incidence of oliguria (24) but not NEC nor bowel perforation. Prophylactic indomethacin is not associated with adverse cognitive or motor outcomes at 36 months corrected age (38) or adverse neurodevelopmental function at 54 months corrected age (39). In our center, due to the risk factors we have identified, and in order to decrease the incidence of severe IVH and minimize potential side effects, we are proposing the use of targeted prophylactic indomethacin to neonates born at <27 weeks, who did not receive a full two doses of antenatal steroids.

### Strengths and Limitations

The main strengths of our study are the prospective completeness of our data collection into the database, which captured 495 of 500 admissions during the period of study, as well as the independent review of cardiac and head ultrasounds images. We were able to retrospectively analyze early cardiac imaging obtained in 52% of participants, which provided additional clinical context. The main limitations of this study relate to our imaging: (1) we do not have early head imaging (in the first 72 h) to delineate the timing of the IVH occurrence with the PDA significance; and (2) we did not have a protocol for serial cardiac ultrasounds at standard time points, so it is possible that some PDAs that were classified as “non-PDA physiology” or “no PDA” actually became hemodynamically significant later within the IVH critical window. Additionally, the definition of PDA significance remains controversial in the neonatal community, and so our definition has its limitations, which may have contributed to the differences seen between our primary and secondary analysis. Finally, this was a prospectively collected and retrospectively analyzed, single-center study, which cannot infer causality, as opposed to a randomized control study.

## Conclusion

Risk factors for IVH were identified and should be the target of IVH reduction bundles. A PDA requiring treatment was associated with a higher risk of IVH. There was no difference in the incidence of IVH in neonates who received medical treatment for PDA closure in the first 72 h of life or had a hs-PDA on imaging in the first 72 h of life, however, early indomethacin treatment for a clinically significant PDA reduced the severity of IVH.

## Data Statement

Individual de-identified participant data can be shared.

## Data Availability Statement

The raw data supporting the conclusions of this manuscript will be made available by the corresponding author on request.

## Ethics Statement

This study was approved by the University of Calgary Conjoint Health Research Ethics Board (CHREB). Data was collected prospectively, using predefined variables, as part of a local quality improvement project aiming at IVH prevention (Drive to Zero), also approved by the CHREB (REB14-1466).

## Author's Note

Some of the information included in this manuscript was previously presented in a poster at the Pediatric Academic Societies Meeting in Toronto, Ontario, Canada (May 7, 2018).

## Author Contributions

IK-L and KM contributed conception, design of the study, and wrote the first draft of the manuscript. KM organized the database and supervised the study. KM and ASo performed the statistical analysis. ASo, ASt, EA, ST, PM, MK, JS, and KM contributed to the acquisition and analysis of imaging data. All authors contributed to manuscript revision, read, and approved the submitted version.

### Conflict of Interest

The authors declare that the research was conducted in the absence of any commercial or financial relationships that could be construed as a potential conflict of interest.

## Abbreviations

IVH: Intraventricular hemorrhage
PHVD: post-hemorrhagic ventricular dilatation
PDA: Patent ductus arteriosus
hs-PDA: Hemodynamically significant patent ductus arteriosus
BPD: Bronchopulmonary dysplasia
NEC: Necrotizing enterocolitis
NSAIDs: Non-steroidal anti-inflammatory drugs
NICU: Neonatal intensive care unit
TNE: Targeted neonatal echocardiogram
IVH-BIPP: Intraventricular Hemorrhage and Brain Injury Prevention Package
RDS: Respiratory Distress Syndrome.
