# Supplementary material for: Intraventricular Hemorrhage: Risk Factors and Association With Patent Ductus Arteriosus Treatment in Extremely Preterm Neonates
Source: Front Pediatr. 2019 Oct 22;7:408. doi: 10.3389/fped.2019.00408 (PMC6817605; doi:10.3389/fped.2019.00408)
Supplement: Supplementary file 1 [file Data_Sheet_1.PDF]

Intraventricular Hemorrhage and Brain Injury Prevention Package

IVH Prevention Working Group

Division of Neonatology

Calgary, AB

November 22, 2017

Table of contents:

1. Background and Resources
2. Lighting
3. Noise
4. Thermoregulation
5. Skin care
6. Minimal Handling
7. Vascular Access
8. Positioning
9. Respiratory Management
10. Hemodynamic Management
11. References
12. Appendix 1

Points of emphasis:

The interventions proposed in this bundle can be divided into two categories:

- Interventions have potential direct impact on IVH (like hemodynamic and respiratory management)
- Interventions have indirect impact on IVH (like light, noise, thermoregulation, minimal handling, and vascular access)
- Minimal handling shouldn't compromise clinical care especially when required to make a care plan

This package is meant to target the neonatal population of less than or equal to $31^{6/7}$ weeks gestation in the first 72 hours of life

1. Background and Resources:

1.1 Background:

Intraventricular hemorrhage (IVH) is a significant cause of moderate-severe brain injury in preterm infants. The majority of IVH occurs within first 72 hrs of life. It has been a clinical challenge to avoid causing injury to the immature brain while balancing management of the very sick premature neonate. There is national and international interest in decreasing the prevalence of IVH. The EPIQ III goal is to drive IVH incidence to zero.

1.2 Resources:

This bundle was put together by IVH working group based on:

a) Literature review

b) Two site visits to BC Women & Children NICU and McMaster NICU

c) Clinical Practice Guidelines (CPG) including- central vascular access, natural cord clamping, age appropriate care, endotracheal suctioning, hypoglycemia, hyperglycemia, intubation, pain, resuscitation and immediate care, thermoregulation, ventilation, very preterm admission.

d) Systematic review of the head scans images

e) Local practice audits:

   (i) 42 cases of severe IVH reviewed systematically by a group of neonatologists

   (ii) Case control audit for 2012

   (iii) Central line insertion audit 2014

   (iv) Intubation audit 2014

   (v) Incidence of temperature fluctuation 2014

   (vi) Incidence of hypoglycemia or hyperglycemia 2014

f) Expert opinions:

   (i) National IVH working group

   (ii) Calgary Neonatal clinical committees (CNS, CVS, Resp, START, and QI)

Back to top

2. Lighting

Light has both positive and negative potential effects on health and development. Our goal is to provide optimal lighting for preterm and ill neonates based on evidence.

Our NICU has a well-defined protocol for environmental lighting. Lighting at all bedsides has been measured and there are ongoing audits to ensure compliance with the lighting CPG.

2.1 Recommendation

For infants ≤31 6/7 weeks and in the IVH Protection Bundle: use continuous dim and indirect lighting; use a penlight when direct visualization is necessary. Following the 72 hours, follow the Neonatal Intensive Care CPG for Environmental Lighting 2-E-4.

2.2 Action

a) Continue with current policy guidelines

2.3 Approach to infants in NICU

a) Maintain continuous dim lighting less than 20 Lux when possible.

b) Use protective eye shield or place a cloth over infant's eyes when direct lighting is necessary.

c) Use indirect lighting (valance or shelf) whenever possible.

d) Use a penlight when direct visualization is necessary. e.g. Monitoring IV/central line/PICC sites/ETT

2.4 Resources required

a) Penlights at every bedside

2.5 Education

a) Handouts to all NICU caregivers

Back to top

3. Noise

3.1 Rationale

The current nationally recognized safe sound levels for the infant rooms in the NICU should not exceed 45 to 50 dB, with transient sound not to exceed 65 to 70 dB. To promote an optimal environment for rest and growth of the neonate, a combination of continuous background and operational sound should be considered.[1]

3.2 Recommendation

a) Recommend noise level less than 45 dB.

3.3 Action

a) Equipment related

(i) Carefully close incubator doors

(ii) Close incubator top as soon as possible

(iii) Handle equipment gently

(iv) Address alarms promptly

(v) Use padded covers on top of the incubator to decrease sound exposure

b) Personnel related

(i) Keep all necessary conversations low and limit conversations at bedside

(ii) All cell phones in NICU should be on vibrate

(iii) Pagers (with the exception of the code pagers) must be on vibrate

(iv) Refrain from placing hard items/writing on the top of the incubator

(v) No loud voices/laughing in patient care areas

(vi) Move rounds away from the bedside in < 31 6/7 week babies for first 72 hours

c) Environment

(i) Admit to “nitric room/D pod” with double doors closed whenever possible. Protect the admit zone from other disruptive admissions/activities.

(ii) Implement a “quiet time” twice daily when staff endeavor to restrict all noise, care procedures, and tests to allow for uninterrupted

sleep time for the infants (quiet time in process of implementation by the Neonatal Age Appropriate Care Committee (NAACC)

(iii) Post signs to identify quiet zone

(iv) Continue to work with NAACC to reduce noise in the NICU.

3.4 Resources required

a) laminated ‘Quiet Zone’ signs

b) Policy change regarding ‘Quiet time’ during day/night shift

3.5 Education

a) Handouts/information/pod talks to all NICU caregivers, educate all care givers of guideline and expectations.

4. Thermoregulation

4.1 Rationale

Both hypothermia and hyperthermia are detrimental to the baby. Hypothermia, particularly in preterm babies, is associated with increased morbidity and mortality. Hyperthermic babies have a worse short-term outcome and can be particularly detrimental in association with intrapartum asphyxia and infection.

4.2 Recommendation

a) Maintain the infant’s temperature at 36.5-37.5°C by servo control adjustment of the incubator environmental temperature.

Action: see Golden Hour Checklist and Thermoregulation Policy 2-T-3

Back to top

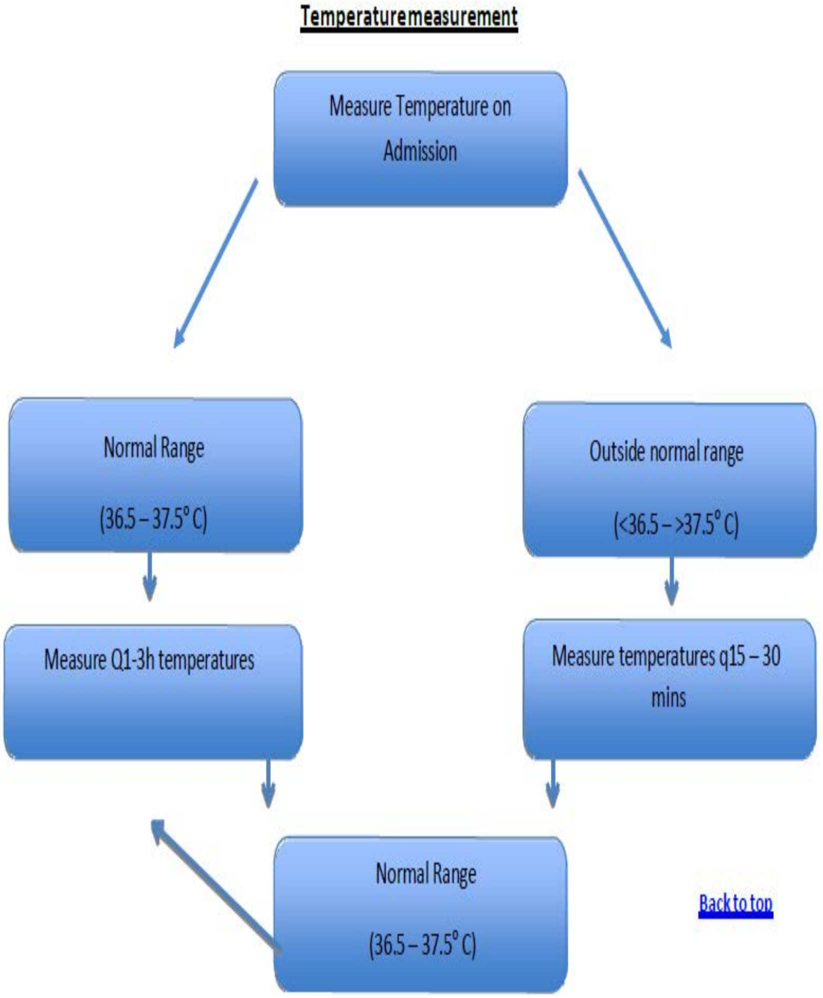

5. Skin Care

5.1 Rationale

Newborns undergo adaptation from the aquatic in utero environment to the aerobic environment after delivery. During this process, their skin assists in the process of thermoregulation, functions as a barrier against toxins and infections, facilitates water and electrolyte balance and serves as a reservoir for fat storage and insulation. It also mediates tactile sensation.

5.2 Recommendation

a) Reduce traumatic injury to skin

5.3 Action

a) Preventing traumatic Injury and dryness

(i) Use only hydrogel electrodes

(ii) Limit use of adhesives

(iii) No bandages on heels, use gentle pressure with sterile gauze to prevent bleeding

(iv) If necessary, loosen adhesives with water based emollients

(v) Use aqueous chlorhexidine with caution for disinfecting surfaces before invasive procedures and always remove disinfectants with warmed sterile water or saline post procedure

(vi) Do not remove vernix caseosa

(vii) No antiseptic soap or cleansers

(viii) No bathing of infants ≤ 31 6/7 weeks

(ix) Provide humidity as per unit guideline

6. Minimal Handling

Minimal manipulation refers to a grouping of care in which the infant is seen as the center of care and requires caregivers to act together, prioritizing the needs of the infant

6.1 Handling can be divided into 4 categories

a) Monitoring: Placement, adjustment and removal of continuous monitoring devices

b) Therapeutic: Umbilical catheters, peripheral IVs, ET insertion and fixation, ET suction, NG/OG insertion, IM injections, Blood letting

c) Nursing: diaper change, skin and eye care, infant positioning, temperature monitoring.

d) Parental: Touching

6.2 Recommendation

Develop/identify a core group of RN’s and RRT’s with specialized knowledge and skills to admit and care for infants ≤31 6/7 weeks for the first week of life.

a) In the first 72 hours the intention is to complete most of the invasive therapeutic interventions and monitoring interventions within the first hour after birth and thereafter only on an ‘as needed’ basis.

b) Any one-care cluster must not exceed 10-15 minutes based on infant's tolerance of handling.

c) Facilitated tucking by second caregiver/parent should be provided during handling whenever possible

d) When changing diapers and performing abdominal girth measurements: lift infant gently by the hips, not the ankles

6.3 Action

a) Minimize newborn examinations

Newborn Examination: Examinations must be restricted to one at the time of admission. If examinations are required thereafter, targeted examinations must be performed only if there is a clinical need.

(i) Recommendation

- 1 standard examination on admission (Follow the examination checklist)
- Do not check for red-reflex and pupillary responses in the 1st week of life.
- CNS examinations should not include primitive reflexes
- Hip assessments should be deferred on ≤ 31 6/7 week infants until before discharge or earlier if clinically indicated.
- Measure only one blood pressure on admission (left arm). Continue with BP measurement as per monitoring policy.
- Daily examinations in infants should always be coordinated with scheduled nursing interventions
- No length measurement for at least the first 72 hours,
- No routine abdominal girth examinations unless ordered by a physician
- No parent skin to skin holding in first 72 hours for intubated babies. Encourage parent/infant skin to skin contact through touching/facilitated tucking.

b) Minimize routine care

(i) Endotracheal (ET) suctioning: Avoid routine suctioning in all infants.

- ET suctioning is commonly associated with a serious fall in transcutaneous oxygen tension. The resultant hypoxemia may

be associated with a rise in blood pressure and increased cerebral blood flow velocity. Consider the schedule of suctioning for each infant individually and continuously review this schedule.

(ii) Temperature assessment

- Continuous temperature assessment must be via Servo-control. Intermittent verification of the Servo function must be performed by manually checking an axilla temperature Q1-3H.

(iii) Intravenous Vitamin K

- Intramuscular Vitamin K avoided in all ≤ 31 6/7 week infants, intravascular injections can be given.

(iv) Regulate Blood Tests

- Obtain standard admission blood tests, other blood tests in the first 72 hours must be ordered in consultation with the neonatologist.

(v) Standards for Cluster care

- Cluster care is simply doing as much as tolerated by the infant (diaper, feeding, suctioning, repositioning, holding, etc.) at one time which allows neonates to have extended amounts of rest.

c) Initial Handling

*Refer to Golden Hour Checklist.

Complete only necessary admission assessments and interventions within the first hour of life. Handling must be brief and gentle. Avoid oral gastric suction unless clinically indicated

(i) Nursing care

- Organize infant care based on infant clinical status and tolerance of handling. Every care cluster/event should be in conjunction with and followed by a 5 minute positive facilitated tucking*.

*Facilitated tucking: the caregiver "hand-swaddling" the infant by placing a hand on the infant's head and feet while providing flexion and containment[2]

- A laminated sign stating in bold letters “Critical Time-Minimal Handling” placed at the baby’s bedside for 72 hours to remind all team members of the protocol.

Back to top

*See appendix 1 for Indications for hands on care

(ii) Initial X-Ray

X-ray must be taken as soon as possible after ETT, central lines and OG placement. The aim is for X-ray to be taken PRIOR TO the administration of surfactant unless the FiO2 on admission is ≥60% then CXR should be done first. Once ETT position confirmed, surfactant administration and line insertion starts.

(iii) Lumbar Puncture

Points of emphasis:

- The majority of ELBWs do not require a lumbar puncture as part of their initial evaluation for early onset sepsis. For example, we should not be doing LPs on ELBW infants solely for maternal risk factors such as PPROM

- The aim is to avoid unnecessary LPs in infants who do not need them but to perform LPs in those infants at highest risk for sepsis/meningitis and have it completed prior to initiation of antibiotics.

- In neonates who are critically ill and likely to have significant cardiovascular or respiratory compromise from the procedure or those with severe thrombocytopenia, the lumbar puncture can be deferred until the infant is more stable.

- The decision not to perform an LP should be regarded as an "active" one and should be documented in the chart. If there is a change in the indications (as above) the LP can still be done 1-2 days after the onset of infection and treatment

6.4 Recommendation

Consider doing an LP only in the following clinical situations:

a) infants with a high probability of sepsis on the basis of clinical signs and/or abnormal lab values

b) infants with positive blood cultures

c) Infants who do not respond to appropriate antimicrobial therapy in the expected manner.

6.5 Rationale

a) The risks associated with handling these vulnerable babies in the first 72 hours of life may outweigh the benefits unless the LP is strongly indicated.

Back to top

7. Vascular Access

*Refer to the Golden Hour Checklist

7.1 Recommendation

a) Establish secure vascular access as early as possible with the least amount of handling /pain for infant.

b) Our aim is to establish vascular access within 30 minutes; prior to the administration of Surfactant unless the FiO2 on admission is ≥60% then CXR should be done first. Once ETT position confirmed, line insertion starts and surfactant administered.

c) Commence IV fluids soon after establishment of access (before CXR) to prevent hypoglycemia.

d) Refrain from using infant restraints for line placements. Infants should be gently contained by a helper.

7.2 Action

a) All umbilical lines should be placed by a senior practitioner who is proficient at umbilical catheter insertions.

b) PIV to be inserted by experienced caregiver only

8. Positioning

8.1 Rationale

Changes in cerebral blood flow and cerebral blood volume can be measured with head in midline and head rotated to the side. It has been observed that the mean cerebral blood volume was significantly higher when the head was rotated 90 degrees to the side. Rotation of the head to one side obliterates the internal jugular vein on the same side.[3] It has also been noted that intracranial pressure is lower when the head is elevated by 30 degrees.[4] Positioning of the infant's head is especially important for critically ill neonates, and all neonates <26 weeks[5]

8.2 Recommendation

a) Maintain neutral head position (head/neck in alignment with body) when turning and positioning the infant with the head of bed elevated 20- 30 degrees.

b) No prone positioning for at least 72 hours if infant is intubated. Infants on CPAP may require prone positioning. Discuss with clinician if appropriate.

c) Positional changes must always be a 2 person job in all infants in the first 72 hours

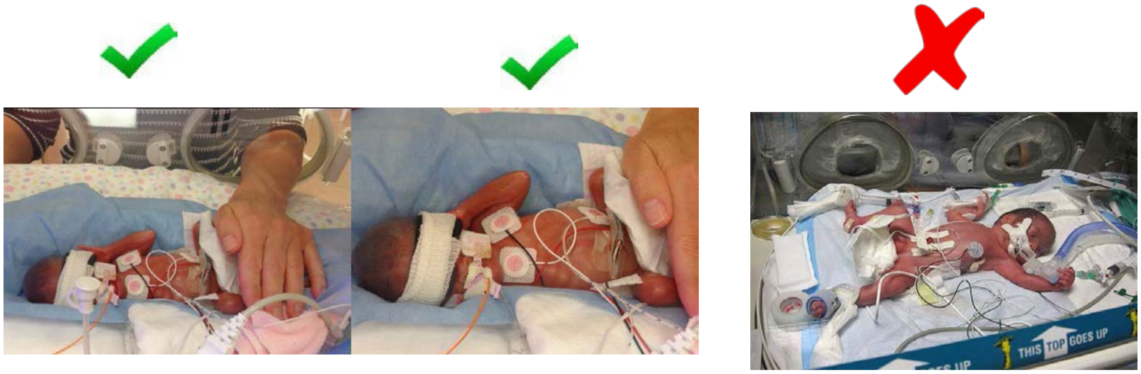

9. Respiratory Management

9.1 Case room resuscitation:

a) Rationale

(i) Intensive resuscitation in the case room may be a risk factor for IVH and brain injury [6]. Effort should be made to ensure resuscitation is as smooth and systematic as possible.

b) Recommendation

*Refer to START Resuscitation Checklist and Optimal Lung Algorithm

(i) The most experienced staff person will be in charge of maintaining the infant airway

(ii) One nurse should be assigned for documentation

(iii) Use the electronic log sheet

(iv) Recruit the lungs by Neopuff CPAP/ventilation prior to intubation (if indicated) following the DR Intubation algorithm.

(v) If possible give the surfactant in the NICU following tube confirmation by xray.

9.2 First 72 hours of life:

a) Rationale

Intubation is a traumatic procedure and evidence shows that brain perfusion and function may be compromised during the intubation.[7] Our audit showed that babies with severe IVH were more frequently reintubated in the first 72 hours of age and CO2 fluctuation was significantly more frequent in the IVH group. There is growing evidence that extreme levels of PCO2 (whether high or low) or fluctuation can increase the risk of brain injury [8, 9] [10-14].

b) Recommendation

If the baby is intubated in the first 72 hours of age:

- ✓ ≤ 24 6/7 weeks GA: keep the infant intubated during the first 72 hours of life. Use protective ventilation strategies to avoid PCO2 fluctuations (especially hypercapnia)
- ✓ ≤25 6/7 -≤ 28 6/7 weeks GA: minimize extubation failure in the first 72 hours of life by ensuring that the baby meets extubation criteria outlined by the treating team or the unit guidelines

(i) Use Ventilation strategies as per optimal lung group recommendations

(ii) PCO2 target of (45-55 mm Hg)[15]

(iii) Have documented criteria for reintubation

(iv) Infants on CPAP should have interface change at least q6h and hat change q24h for the first 72 hours. Resume care as per unit policy following the 72 hours

Back to top

10. Hemodynamic Management:

10.1 PDA

a) Rationale

Hemodynamically significant PDA is a risk factor for IVH[16] [17]. Our audit showed babies with severe IVH had significantly more PDA ligations later in life. We also found no baby in IVH group was treated for PDA in the first 72 hours of age. This may be an indication for recognizing and treating hemodynamically significant PDA at an earlier stage. There is evidence that early treatment increases the PDA closure rate[18-21].

b) Recommendation

For intubated babies:

(i) TNE at 24- 48 hours of age (unless clinically indicated earlier) to identify hemodynamically significant* PDA

(ii) TNE time should as short as possible

(iii) It is not recommended to treat bidirectional PDA as it might have protective effect by preventing cerebral ischemia that occurs due to steeling phenomenon in left to right shunting PDA. Treating the PDA may put extra strain on the right ventricle as the PDA may help as a pop off valve in the presence of pulmonary hypertension

(iv) Consider treatment with indomethacin :

- ✓ ≤ 25 6/7 weeks GA: if the PDA is hemodynamically significant*
- ✓ ≤26 6/7- ≤28 6/7weeks GA: if intubated, PDA is hemodynamically significant*, and impacting ventilation

*Hemodynamically significant PDA[22]:

- Size ≥ 1.5 mm
- Flow pattern: pulsatile or low velocity
- Flow direction: left to right
- LA/AO ratio ≥1.5
- Pseudo normalization of mitral inflow velocity (E/A >1)
- Decreased or absent diastolic flow in middle cerebral artery, celiac/superior mesenteric artery

10.2 Hypotension

a) Rationale

Our audit and other studies has shown that use of inotropes is a significant risk for severe IVH [11, 23-25] and white matter injury [26]. However, we do not have a standard definition for hypotension or a standardized approach to the management of hypotension [27]. We know that infusing inotropes (especially dopamine) in an infant brain with compromised auto-regulation, will change the curve to pressure passive and exaggerate reperfusion injury like IVH. [23]

b) Recommendation

(i) Natural cord clamping when feasible

(ii) Avoid the use of inotropes if possible. Consider inotrope use only if there are at least 2 or more of the following criteria associated with low BP:

- Lactate > 3.5 mmol/l
- CRT > 3 seconds
- Urine output < 1 ml/kg/hour (beyond 24 hours of age)
- Echocardiography shows low cardiac output and/or low SVC flow

(iii) Before commencing Inotropes consider:

- Rule out hyperinflation and iatrogenic hypotension/raised SVC pressure
- Fluid status / potential dehydration
- A fluid bolus administered slowly over at least 30 minutes

(iv) Consider other markers for hemodynamic instability in addition to Blood pressure in hypotension management such as:

- Urine Output (beyond 24 hours of age)
- Capillary refill time
- Heart rate trends
- Oxygen saturation
- Metabolic acidosis
- Lactic acidosis

(v) After initiation of inotropes, allow 30-60 minutes for infant to respond to the medication before increasing dose.

(vi) Avoid sodium bicarbonate or THAM infusion [28, 29]

(vii) Accept arterial pH ≥ 7.20, provided PCO2 within target range

10.3 Other

a) Thrombocytopenia

Platelet transfusions may be used to lower the risk of serious hemorrhage in a sick, preterm neonate with thrombocytopenia. Generally, the preterm and sick infant should have a platelet count greater than 50 X 109/L) [22].

b) Hypo/Hyperglycemia

Euglycemia is desired. The target glucose range is greater than 2.6 mmol/L and less than 12 mmol/L.

c) Electrolytes

Both sodium and potassium values should be monitored with a goal to maintain the electrolytes in normal range.

Back to top

11. References

1. Graven, S.N., Sound and the developing infant in the NICU: conclusions and recommendations for care. J Perinatol, 2000. 20(8 Pt 2): p. S88-93.

2. Ward-Larson, C., R.A. Horn, and F. Gosnell, The efficacy of facilitated tucking for relieving procedural pain of endotracheal suctioning in very low birthweight infants. MCN Am J Matern Child Nurs, 2004. 29(3): p. 151-6; quiz 157-8.

3. Pellicer, A., et al., Noninvasive continuous monitoring of the effects of head position on brain hemodynamics in ventilated infants. Pediatrics, 2002. 109(3): p. 434-40.

4. Emery, J.R. and J.L. Peabody, Head position affects intracranial pressure in newborn infants. J Pediatr, 1983.103(6): p. 950-3.

5. Ancora, G., et al., Effect of posture on brain hemodynamics in preterm newborns not mechanically ventilated. Neonatology, 2009. 97(3): p. 212-7.

6. Duerden, E.G., et al., Resuscitation intensity at birth is associated with changes in brain metabolic development in preterm neonates. Neuroradiology, 2013. 55 Suppl 2: p. 47-54.

7. van den Berg, E., et al., Effect of the "InSurE" procedure on cerebral oxygenation and electrical brain activity of the preterm infant. Arch Dis Child Fetal Neonatal Ed, 2009. 95(1): p. F53-8.

8. Noori, S., et al., Effect of carbon dioxide on cerebral blood flow velocity in preterm infants during postnatal transition. Acta Paediatr, 2014. 103(8): p. e334-9.

9. Noori, S., et al., Changes in cardiac function and cerebral blood flow in relation to peri/intraventricular hemorrhage in extremely preterm infants. J Pediatr, 2013. 164(2): p. 264-70 e1-3.

10. Kaiser, J.R., et al., Hypercapnia during the first 3 days of life is associated with severe intraventricular hemorrhage in very low birth weight infants. J Perinatol, 2006. 26(5): p. 279-85.

11. Khodapanahandeh, F., N. Khosravi, and T. Larijani, Risk factors for intraventricular hemorrhage in very low birth weight infants in Tehran, Iran. Turk J Pediatr, 2008. 50(3): p. 247-52.

12. Fabres, J., et al., Both extremes of arterial carbon dioxide pressure and the magnitude of fluctuations in arterial carbon dioxide pressure are associated with severe intraventricular hemorrhage in preterm infants. Pediatrics, 2007. 119(2): p. 299-305.

13. Erickson, S.J., et al., Hypocarbia in the ventilated preterm infant and its effect on intraventricular haemorrhage and bronchopulmonary dysplasia. J Paediatr Child Health, 2002. 38(6): p. 560-2.

14. Shankaran, S., et al., Cumulative index of exposure to hypocarbia and hyperoxia as risk factors for periventricular leukomalacia in low birth weight infants. Pediatrics, 2006. 118(4): p. 1654-9.

15. Hagen, E.W., et al., Permissive hypercapnia and risk for brain injury and developmental impairment. Pediatrics, 2008. 122(3): p. e583-9.

16. Kim, K.R., S.W. Jung, and D.W. Kim, Risk factors associated with germinal matrix-intraventricular hemorrhage in preterm neonates. J Korean Neurosurg Soc, 2014. 56(4): p. 334-7.

17. Sellmer, A., et al., Morbidity and mortality in preterm neonates with patent ductus arteriosus on day 3. Arch Dis Child Fetal Neonatal Ed, 2013. 98(6): p. F505-10.

18. Varvarigou, A., et al., Early ibuprofen administration to prevent patent ductus arteriosus in premature newborn infants. Jama, 1996. 275(7): p. 539-44.

19. Knight, D.B., The treatment of patent ductus arteriosus in preterm infants. A review and overview of randomized trials. Semin Neonatol, 2001. 6(1): p. 63-73.

20. Van Overmeire, B., et al., Early versus late indomethacin treatment for patent ductus arteriosus in premature infants with respiratory distress syndrome. J Pediatr, 2001. 138(2): p. 205-11.

21. Kluckow, M., et al., A randomised placebo-controlled trial of early treatment of the patent ductus arteriosus. Arch Dis Child Fetal Neonatal Ed, 2014. 99(2): p. F99-F104.

22. McNamara, P.J. and A. Sehgal, Towards rational management of the patent ductus arteriosus: the need for disease staging. Arch Dis Child Fetal Neonatal Ed, 2007. 92(6): p. F424-7.

23. Lightburn, M.H., et al., Observational study of cerebral hemodynamics during dopamine treatment in hypotensive ELBW infants on the first day of life. J Perinatol, 2013. 33(9): p. 698-702.

24. Kuint, J., et al., Early treated hypotension and outcome in very low birth weight infants. Neonatology, 2009.95(4): p. 311-6.

25. Pekcevik, Y.M., et al., Risk Factors of Germinal Matrix Intraventricular Hemorrhage in Premature Infants. Iran J Pediatr, 2014. 24(2): p. 191-197.

26. Mohammad, k. (2012) Hypotension and White Matter Injury (WMI) in Preterm Neonates. Volume,

27. Wong, J., et al., Inotrope Use among Extremely Preterm Infants in Canadian Neonatal Intensive Care Units: Variation and Outcomes. Am J Perinatol, 2014. 32(1): p. 9-14.

28. Papile, L.A., et al., Relationship of intravenous sodium bicarbonate infusions and cerebral intraventricular hemorrhage. J Pediatr, 1978. 93(5): p. 834-6.

29. Berg, C.S., et al., Sodium bicarbonate administration and outcome in preterm infants. J Pediatr, 2010. 157(4): p.684-7.

Back to top

Indications for hands on care of the ≤31 6/7 week infant
*Accompanied by facilitated tucking for infant throughout:

Admission:

- Airway established and effective ventilation
- Weight (if not done in delivery room)
- Place on blue burn sheet
- Attach bedside SpO2 monitor to probe attached to infant in delivery room
- Skin temperature probe attached to infant on right/left lower back inside the diaper (probe works best with infant lying on it. May need 2 probes attached; one on each side and use probe from appropriate side of infant to monitor temp)
- Axilla temperature
- Place chest leads on and attach to monitor
- BP per one upper limb (left)
- Obtain POCT glucose if central lines not inserted by 30 minutes of age
- One admission exam by physician/NP coordinated with bedside RN

Ongoing care:

- limit 10-15 minutes of handling accompanied by facilitated tucking throughout
- Obtain as much information as possible from observation
- "Top down" and 90% humidity once central lines inserted
- Aquaphor SPARINGLY and avoid areas where temperature skin probe and ECG leads applied
- Monitor dryness of blue burn sheeting with each handling of infant and change prn
- Change diaper and monitor output q4-6h
- Reposition infant supine or side-lying q4-6H (as a 2 person task)
- Assess central line placement q1h through observation
- Assess BP continuously through UAC or obtain peripheral BP as needed.
- Assess axilla temperature q1-3h as per policy. Continuously monitor isolette air temperature and how well skin probe attached and reading to determine stability of infant temperature
- POCT glucose as per unit policy
- Assess PIPP score with each handling
- Respiratory assessments as needed in conjunction with physician/NP/RRT
- Assess fontanels, activity, tone, heart sounds, air entry, apex, perfusion, peripheral pulses, abdomen, skin as needed while maintaining a 10-15 minute handling maximum and use of a second caregiver for facilitated tucking of infant

*Care should be infant cue based.

Back to top
