# Supplementary material for: Intraventricular Hemorrhage: Risk Factors and Association With Patent Ductus Arteriosus Treatment in Extremely Preterm Neonates
Source: Front Pediatr. 2019 Oct 22;7:408. doi: 10.3389/fped.2019.00408 (PMC6817605; doi:10.3389/fped.2019.00408)
Supplement: Supplementary file 2 [file Data_Sheet_2.PDF]

Alberta Health Services

GUIDELINE

TITLE

INTRAVENTRICULAR HEMORRHAGE AND BRAIN INJURY PREVENTION ON HIGH RISK NEONATAL TRANSPORT

**SCOPE**<br>Neonatal Intensive Care, Calgary Zone	**DOCUMENT #**<br>2-I-10
**APPROVAL AUTHORITY**<br>Calgary Neonatal Care Committee	**INITIAL EFFECTIVE DATE**<br>September 27, 2016
**SPONSOR**<br>Neonatal Intensive Care/Division of Neonatology, Calgary Zone	**REVISION EFFECTIVE DATE**<br>November 17, 2016
**PARENT DOCUMENT TITLE, TYPE AND NUMBER**<br>Not applicable	**SCHEDULED REVIEW DATE**<br>September 27, 2021

NOTE: The first appearance of terms in bold in the body of this document (except titles) are defined terms – please refer to the Definitions section.

If you have any questions or comments regarding the information in this document, please contact the Policy & Forms Department at policy@ahs.ca. The Policy & Forms website is the official source of current approved policies, procedures, directives, standards, protocols and guidelines.

OBJECTIVES

- To promote a safe and consistent standard of care for all high risk neonatal transports potentially at risk for Intraventricular Hemorrhage (IVH) and/or brain injury. Current literature supports the establishment of clear guidelines on additional care measures to practice when transporting at risk neonates.
- Clinical judgment may be exercised when a situation is determined to be outside the parameters provided in this guideline. If a deviation from this guideline is determined to be appropriate or necessary, documentation of the rationale shall be included on the patient's health record.

PRINCIPLES

Inclusion Criteria

- o Neonates requiring transport that are both:
  - Less than or equal to $30^{+6/7}$ weeks gestational age [1, 2, 3]
  - Within the first 72 hours of life [1, 4]

In-Utero Transport

- o Transportation of a very low birth weight neonate may increase the risk of IVH. Therefore, it is recommended to transfer patients in-utero as per network guidelines to a tertiary care centre (Foothills Medical Centre) specializing in high-risk deliveries of preterm newborns. [4, 1, 5, 3, 6]

- If in-utero transport is not possible, the NICU transport team should make every possible attempt to arrive at the sending site prior to delivery. Intensive resuscitation (low 5 minute Apgar) in the delivery room may be a risk factor for IVH and brain injury. Therefore, having skilled and experienced staff on hand is recommended to ensure the delivery is as smooth and systematic as possible. [7, 8, 9, 2]
    - The safest and fastest mode of transport (ground or air) should be arranged for the arrival of the NICU transport team.
    - It is ideal to have additional trained personnel skilled in intubation and emergency line insertion to attend the delivery of preterm neonates. [2, 5]
        - Have the most experienced personnel onsite manage airway/Intubate as per the Intubation guideline. (2-I-3) [10]
        - Follow NRP resuscitation algorithm for low birth weight babies [2]
            - Start on 30% FiO2 and monitor SpO2 to NRP targets.
            - Attempt CPAP +5 cmH2O for the spontaneous breathing infant. [27]
            - If required, give PPV at an initial PIP of 20-25cmH2O.
                - Use the lowest amount pressure to achieve a rise in heart rate (greater than 100bpm) with adequate chest expansion.
            - Excessive PPV or CPAP during resuscitation increases IVH risk by
                - Decreasing venous return
                - Increasing risk of pneumothorax
            - Resuscitation should be performed on a flat surface.
            - Use polyethylene bag for all babies in this guideline. [27]
            - Use chemical heating mattress on radiant warmer
            - Consider having Surfactant replacement medication available to administer to babies less than or equal to 29^{+6} weeks gestation. [27]
        - Recommend natural cord clamping for 60 seconds for infants who do not require immediate resuscitation. Refer to guideline 2-N-4: Natural Cord Clamping)

APPLICABILITY

Compliance with this document is required by all Alberta Health Services employees, members of the medical and midwifery staffs, Students, Volunteers, and other persons acting on behalf of Alberta Health Services (including contracted service providers as necessary) Calgary Zone, Neonatal Intensive Care.

ELEMENTS

1. Positioning

1.1 Keep stretcher level whenever possible to prevent Trendelenburg position [2, 11]

1.2 Head midline to shoulders with patient in supine position to prevent obstruction of jugular venous drainage [11, 12, 13]

1.3 Use positioning aids to facilitate midline positioning during stabilization and transportation of neonates.

1.4 Due to multiple manipulations the stretcher goes through for loading and unloading in airplanes and ambulances during transport, we recommend NOT changing the degree of head of bed at this time.

2. Noise/Vibration

2.1 Sound levels experienced by neonates should not exceed 45 dBA as per guideline Noise Levels: 2-N-3 [14, 15]

a) Neonatal transports exceed these levels as soon as the incubator leaves the NICU with readings of 60-80 dBA. These levels have been associated with increases in intracranial pressure which could potentially increase the risk for IVH. [14, 15]

(i) A closed isolette will only reduce sound levels by 6 dBA [15]

(ii) No additional incubator modifications have been found effective at further reducing sound levels [14]

b) Sound exposure to the neonate must be limited by reducing the sound that reaches the individual neonate by using earmuffs or ear plugs [15]

(i) If skin integrity allows, provide ear protection to the infant. Hydrogel adhesive ear protection is recommended for transport use, and reduces sound by 7 dBA.

(ii) If skin integrity is questionable or compromised, a cotton ball can be used to remove adhesive from the ear protection device. The ear protection can then be secured under the hat and over the ears.

2.2 Transportation potentially exposes neonates to intense vibrational forces not experienced in the classic NICU setting. Neonates of this age have not

developed the ability to counterbalance these forces. Vibration exposure may induce an inflammatory reaction which has been implicated in worsening IVH. [16]

a) Standard foam mattresses are effective in reducing vibration during transportation of infants at a tested weight of 2kgs. [14]

(i) A gel mattress alone was less effective in the model with a 300g infant [14]

b) The combination of a standard foam mattress with a gel mattress on top has proven to reduce the amount of vibration exposure to model infants. [14]

(i) Use standard foam mattress with a gel mattress on top.

- You may consider adding chemical heated transport mattress to the gel mattress if required for temperature.

c) Minimize ambulance transport time when available and practical. [14]

(i) Take shortest route at the safest speed for patient care to reduce vibration exposure in the ambulance.

(ii) Utilize the fixed wing transportation to reduce out of hospital time and ambulance vibration exposure.

3. Respiratory Management

3.1 When applicable, manage patient as per guideline: Nasal Continuous Positive Airway Pressure (NCPAP) on Transport (2-C-15)

3.2 If required, have the most experienced personnel onsite manage airway/Intubate as per intubation guideline (2-I-3) [10]

a) Chest X-ray confirmation for ETT depth and ventilation assessment

b) Consultation with attending neonatologist is REQUIRED prior to surfactant replacement therapy.

3.3 Permissive hypercapnia is a safe and effective way to utilize a lung protective ventilation strategy to achieve a desired CO2 range. Fluctuations of CO2 values outside of this range have been associated with an increased risk of developing IVH. [17, 18, 19, 20, 21, 2]

a) Target CO2 range 45 - 55 mmHg [17]

(i) Monitor blood gas values (Consult receiving neonatologist for frequency) [2, 20]

(ii) Use TcCO2 and ETCO2 monitors to trend with blood gas CO2 values [18]

b) Use a ventilator that allows for a synchronized ventilation mode [22]

(i) IPPV ventilator may increase the difficulty of maintaining CO2 targets [18]

4. Lighting

4.1 As per the NICU policy Environmental Lighting (2-E-4) dimmed lighting is ideal for all babies. Also the infant's eyes should not be exposed to direct light.

a) NICU transports may involve natural light exposure and/or staff safety lighting in excess of what the infant would be exposed to in a NICU environment.

(i) Use isolette cover

(ii) Place a small blanket over the eyes to shield light

b) Use a pen-light for direct visualization when necessary (ie. ETT markings/IV sites)

c) Defer eye exam for red reflex.

d) Erythromycin can be applied without eye exam.

5. Thermoregulation

5.1 Hypothermia is associated with increased morbidity and mortality, especially in preterm babies. Hyperthermia causes worse short-term outcomes and can be detrimental in association with intrapartum asphyxia and infection. Body temperature has been associated as a marginal risk factor for IVH. [1]

a) Maintain infant temperature 36.5-37.5°C

b) Use isolette skin temperature monitoring probe

(i) Trend value with axillary temperature monitoring when necessary

c) Preheat transport isolette to 32-35.4°C [23]

(i) May need to adjust settings according to outside weather conditions

d) Place infant in a food grade, polyethylene bag for transport

e) Place a hat/toque

f) Consider Chemical heated transport mattress during transport

g) Consider placing the infant in a silver swaddler during transport

h) Note: Patient must be at goal temperature when placed in silver swaddler

6. Skin Care

6.1 Infant's skin assists in the process of thermoregulation, functions as a barrier against toxins and infections, facilitates water and electrolyte balance and serves as a reservoir for fat storage and insulation. (2-S-1)

a) Use hydrogel electrodes

b) Limit adhesive bandage use

(i) Use gentle pressure with sterile gauze to stop bleeding if required

c) Loosen adhesives with water based emollients

d) Use 0.5% chlorhexidine and 70% alcohol prior to any skin breaks or invasive line procedures

(i) Remove excess chlorhexidine with warmed sterile water or saline post procedure

7. Hemodynamic Management

7.1 The use of inotropes has been shown to increase the risk of severe IVH. [8, 9, 24, 25] Studies have shown that the infusion of inotropes (especially dopamine) to optimize blood pressure likely impair cerebral autoregulation and increase cerebral blood flow, thus resulting in exaggerated reperfusion injury such as IVH. [24]

a) With the exception of emergency medications; consult with the transport neonatologist for orders and goals for continuing or starting vasopressor therapy.

b) If a fluid bolus is ordered from receiving neonatologist, give slowly over at least 30 minutes [2]

c) Avoid a sodium bicarbonate or hypertonic glucose bolus [2]

d) Avoid unnecessary non-invasive blood pressure measurements

(i) Consider just an upper and lower limb blood pressure [26]

(ii) Keep blood pressure cuff on a selected limb to avoid excess handling

e) In consultation with the receiving neonatologist, consider central line placement for the following gestations

(i) Less than or equal to 26^{+6} weeks place UAC/UVC (double lumen)

(ii) Greater than or equal to 27^{+0} weeks place UVC (double lumen)

8. Minimal Handling

8.1 Minimal manipulation refers to a grouping of care in which the infant is seen as the centre of care and requires caregivers to act together, prioritizing the needs of the infant. Minimal handling is recommended in the management of infants at risk for IVH [22]

a) To minimize stimulation and excessive handling; recommend no skin to skin cuddling with parents after stabilization and prior to transport.

(i) Provide mother with ladybug package and encourage parents to provide facilitated tucking and containment.

b) Attempt to cluster care as much as possible and handle patient gently. [2]

9. Equipment Required

- CossVent Transport Isolette
- Food-grade polyethylene bag
- Hat
- Ear muff set / ear plugs
- Cotton balls
- Penlight
- Eye cover
- Ladybug package
- Gel mattress
- Chemical heated transport mattress
- Head positioning aid
- Silver swaddler

DEFINITIONS

None.

AUTHORS

© Alberta Health Services (AHS)

Luke Petan RRT, Lindsay Moon TRN

REFERENCES

- Alberta Health Services Resources:
  - Neonatal Intensive Care: 2-I-3 Intubation
  - Neonatal Intensive Care: 2-N-4 Natural Cord Clamping – Preterm Infants
  - Neonatal Intensive Care: 2-C-15 Nasal Continuous Positive Airway Pressure (NCPAP) on Transport
  - Neonatal Intensive Care: 2-E-4 Environmental Lighting
  - Neonatal Intensive Care: 2-N-3 Noise Levels
  - Neonatal Intensive Care: 2-S-1 Skin and Wound Care
- Non-Alberta Health Services Documents:
  1. M. Gleibner, G. Jorch and S. Avenarius, "Risk Factors for intraventricular hemorrhage in birth cohort of 3721 premature infants," Journal of Perinatal Medicine, no. 28, pp. 104-110, 2000.
  2. American Heart Association, Neonatal Resuscitation Textbook 6th Edition, American Academy of Pediatrics, 2011, pp. 267-282.
  3. M. A. Mohamad and H. Aly, "Transport of premature infants is associated with increased risk for intraventricular haemorrhage," Arch Dis Child Fetal Neonatal Ed, no. 95, pp. F403-F407, 2010.
  4. M. Hohlagschewandter, P. Husslein, K. Klebermass, M. Weninger, A. Nardi and M. Langer, "Perinatal mortality and morbidity, Comparison between maternal transport, neonatal transport and inpatient antenatal treatment," Archives of Gynecology and Obstetrics, no. 265, pp. 113-118, 2001.
  5. F. Longhini and e. al., "Outcomes of Preterm Neonates Transferred between Tertiary Perinatal Centers," Pediatric Critical Care Medicine, no. 16, pp. 733-738, 2015.
  6. C. Towers, R. Bonebrake, G. Padilla and P. Rumney, "The Effect of transport on the Rate of Severe Intraventricular Hemorrhage in Very Low Birth Weight Infants," The American College of Obstetricians and Gynecologists, vol. 95, no. 2, pp. 291-295, 2000.
  7. E. Duerden, M. Brown-Lum and et al. , "Resuscitation intensity at birth is associated with changes in brain metabolic development in preterm neonates," Paediatric Neuroradiology, no. 55, pp. S47-S54, 2013.
  8. Y. Pekcevik, E. A. Ozer, N. Erdogan and A. Pasinli, "Risk Factors of Germinal Matrix Intraventricular Hemorrhage in Premature Infants," Iran Journal of Pediatrics, vol. 24, no. 2, pp. 191-197, 2014.
  9. F. Khodapanahandeh, N. Khosravi and T. Larijani, "Risk factors for intraventricular hemorrhage in very low birth weight infants in Tehran, Iran," The Turkish Journal of Pediatrics, no. 50, pp. 247-252, 2008.
  10. E. Van Den Burg, P. M. A. Lemmers, M. C. Toet, J. H. G. Klaessens and F. Van Bel, "Effect of the "InSurE" procedure on cerebral oxygenation and electical brain activity of the preterm infant," Arch Dis Child Fetal Neonatal Ed, no. 95, pp. F53-F58, 2010.
  11. J. L. Peabody and J. R. Emery, "Head position affects intracranial pressure in newborn infants," The Journal of Pediatrics, vol. 103, no. 6, pp. 950-953, 1983.
  12. G. Ancora, E. Maranella, A. Aceti, L. Pierantoni, S. Grandi, L. Corvaglia and G. Faldella, "Effect of Posture on Brain Hemodyamics in Preterm Newborns Not Mechanically Ventilated," Neonatology, no. 97, pp. 212-217, 2010.

© Alberta Health Services (AHS)

13. A. Pellicer, F. Gaya, R. Madero, J. Quero and F. Cabanas, "Noninvasive Continuous Monitoring of the Effects of Head Position on Brain Hemodynamics in Ventilated Infants," Pediatrics, vol. 109, no. 3, pp. 434-440, 2002.
14. J. Prehn, I. McEwen, L. Jeffries, M. Jones, T. Daniels, E. Goshorn and C. Marx, "Decreasing sound and vibration during ground transport of infants with very low birth weight," Journal of Perinatology, no. 35, pp. 110-114, 2015.
15. A. Almadhoob and A. Ohlsson, "Sound reduction management in the neonatal intensive care unit for preterm or very low birth weight infants," The Cochrane Collaboration, no. 1, pp. 1-14, 2015.
16. S. Shah, A. Rothberger, M. Caprio, P. Mally and K. Hendricks-Munoz, "Quantification of impulse experienced by neonates during inter- and intra-hospital transport measured by biophysical accelerometery," Journal of Perinatal Medicine, no. 36, pp. 87-92, 2008.
17. E. Hagen, M. Sadek-Badawi, D. Carlton and M. Palta, "Permissive Hypercapnia and Risk for Brain Injury and Developmental Impairment," American Academy of Pediatrics, vol. 122, no. 3, pp. e583-e589, 2008.
18. R. Maheshwari and M. Luig, "Review of Respiratory Management of Extremely Premature Neonates During Transport," Air Medical Journal, no. 33:6, pp. 268-291, 2014.
19. J. Kaiser, C. Gauss, M. Pont and D. Williams, "Hypercapnia during the first 3 days of life is associated with severe intraventricular hemorrhage in very low birth weight infants," Journal of Perinatology, no. 26, pp. 279-285, 2006.
20. J. Forbes, W. Carlo, V. Phillips, G. Howard and N. Ambalavanan, "Both Extremes of Arterial Carbon Dioxide Pressure and the Magnitude of Fluctuations in Arterial Carbon Dioxide Pressure Are Associated With Severe Intraventricular Hemorrhage in Preterm Infants," Pediatrics, vol. 119, no. 2, pp. 299-304, 2007.
21. S. Erickson, A. Grauaug, L. Gurrin and M. Swaminathan, "Hypocarbia in the ventilated preterm infant and its effect on intraventricular haemorrhage and bronchopulmonary dysplasia," Journal of Paediatr. Child Health, no. 38, pp. 560-562, 2002.
22. R. J. Martin, A. A. Fanaroff and M. C. Walsh, "Fanaroff and Martin's Neonatal-Perinatal Medicine: Diseases of the Fetus and Infant," 2015, pp. 890-903.
23. G. Merenstein and S. Gardner, Handbook of Neonatal Intesnive Care (6th Ed), Mosby Elsevier, 2006.
24. M. Lightburn, C. Gauss, D. Williams and J. Kaiser, "Observational study of cerebral hemodynamics during dopamine treatment in hypotensive ELBW infants on the first day of life," Journal of Perinatology, no. 33, pp. 698-702, 2013.
25. J. Wong, P. Shah, E. Yoon, W. Yee, S. Lee and K. Dow, "Inotrope Use among Extremely Preterm Infants in Canadian Neonatal Intensive Care Units: Variation and Outcomes," American Journal of Perinatol, no. 32, pp. 9-14, 2014.
26. N. Patankar, N. Fernandes, K. Kumar, V. Manja and S. Lakshminrusimha, "Does measurement of four-limb blood pressures at birth improve detection of aortic arch anomalies?," Journal of Perinatology, no. 36, pp. 376-380, 2016.
27. Perlman JM, Wyllie J, Kattwinkel J, Wyckoff MH, Aziz K, Guinsburg R, Kim HS. Liley HG, Mildenhall L, Simon WM, Szyld E, Tamura M, Velaphi S: on behalf of the Neonatal Resuscitation Chapter Collaborators. Part 7: neonatal resuscitation: 2015 International Consensus on Cardiopulmonary Resuscitation and Emergency Cardiovascular Care Science With Treatment Recommendations. *Circulation*. 2015:132(suppl 1):S204-S241

VERSION HISTORY

**Date**	**Action Taken**
November 17, 2016	Non-substantive change
Click here to enter a date	Optional: Choose an item
