# Supplementary material for: Intraventricular Hemorrhage: Risk Factors and Association With Patent Ductus Arteriosus Treatment in Extremely Preterm Neonates
Source: Front Pediatr. 2019 Oct 22;7:408. doi: 10.3389/fped.2019.00408 (PMC6817605; doi:10.3389/fped.2019.00408)
Supplement: Supplementary file 3 [file Data_Sheet_3.PDF]

INTRAVENTRICULAR HEMORRHAGE GRADING PRE-TERM NEONATES:

Case: ☐ Normal: ☐ Reviewer Initials (First/Last): ☐

Patient Initials (First/Last): ______________ ID#: ____________________________

#1 – Baseline Scan US (YYYY/MM/DD): __________/ ______/ ______

	Right Brain	Left Brain
IVH Grade:	☐	☐

IVa/b: Right Brain IVa/b: Left Brain

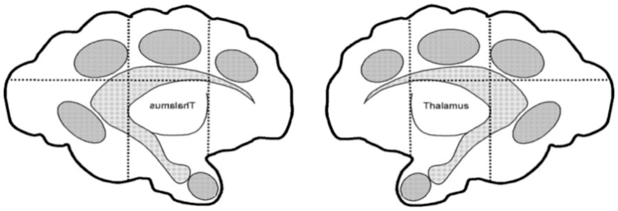

	Right Brain	Left Brain
VI (mm)	☐	☐
AHW (mm)	☐	☐

FOHR (mm) A: ☐ B: ☐ C: ☐

#2 – Hydrocephalus Scan US (YYYY/MM/DD): __________/ ______/ ______

	Right Brain	Left Brain
VI (mm)	☐	☐
AHW (mm)	☐	☐

FOHR (mm) A: ☐ B: ☐ C: ☐

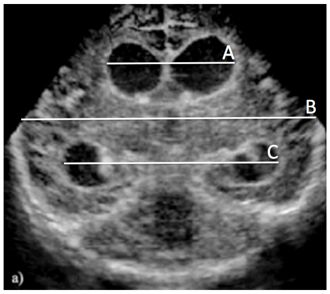

v. 20-Dec-2016

#3 – Discharge Scan US (YYYY/MM/DD): __________/ ______/ ______

	Right Brain	Left Brain				
VI (mm)	☐	☐				
AHW (mm)	☐	☐	FOHR (mm)	A: ☐	B: ☐	C: ☐

Right Brain

Cystic PVL	No ☐	Micro ☐	Macro ☐
Porencephalic Cyst	No ☐	Yes ☐	
Persistent Hydrocephalus	No ☐	Yes ☐	
Resolved IVH	No ☐	Yes ☐	

Left Brain

Cystic PVL	No ☐	Micro ☐	Macro ☐
Porencephalic Cyst	No ☐	Yes ☐	
Persistent Hydrocephalus	No ☐	Yes ☐	
Resolved IVH	No ☐	Yes ☐	

Grading Terms of Engagement:

#1 Baseline Scan: is the CUS performed in first 7 days-of-life that has most severe grade <u>OR</u> if no week one CUS exam performed, then grade the first CUS regardless of date.
#2 Hydrocephalus Scan: is the CUS that demonstrates the most severe degree of post-hemorrhagic ventricular dilatation.
#3 Discharge Scan: is the latest available CUS (using Enterprise search) that was performed at, or before 40 wks CGA.
